# Diagnosis and Treatment of Gastrinomas in Multiple Endocrine Neoplasia Type 1 (MEN-1)

**DOI:** 10.3390/cancers4010039

**Published:** 2012-01-20

**Authors:** Ursula Plöckinger

**Affiliations:** Interdisziplinäres Stoffwechsel-Centrum: Endokrinologie, Diabetes und Metabolismus, Kompetenzzentrum Seltene Stoffwechselkrankheiten, Charité-Universitätsmedizin Berlin, Campus Virchow-Klinikum, Berlin 13353, Germany; E-Mail: ursula.ploeckinger@charite.de; Tel.: +49-30-450-553-552; Fax: +49-30-450-553-950

**Keywords:** MEN-1, gastrinoma, diagnosis, therapy

## Abstract

Multiple endocrine neoplasia type 1 (MEN-1) is a rare autosomal-dominant disease. It is associated with a broad range of endocrine tumours, most frequently arising in the parathyroid glands, the pituitary and the pancreas. Most neuroendocrine tumours will be diagnosed in the pancreas as non-functioning neuroendocrine tumours or insulinomas. Forty-two percent of the patients will develop a gastrin-secreting neuroendocrine tumour, a gastrinoma. Gastrinomas in MEN-1 tend to be small, multiple and preferentially located in the duodenum. This paper will focus on the specific characteristics of gastrinomas in the setting of MEN-1 compared to sporadic gastrinomas. The developments in understanding the tumorigenesis of these tumours and the consequences for diagnosis and therapy will be discussed.

## 1. Introduction

Multiple endocrine neoplasia type 1 (MEN-1) is a rare autosomal-dominant disease. MEN-1 is associated with a broad range of endocrine tumours, most frequently arising in the parathyroid glands, the pituitary and the pancreas. Penetrance for neuroendocrine tumours is up to 70%–100%. Half of the patients with MEN-1 will present with a pancreatico-duodenal neuroendocrine tumour at the age of 50 years. Most will arise in the pancreas as non-functioning neuroendocrine tumours or insulinomas. Forty-two percent (range 20%–61%) of the patients will develop a gastrin-secreting neuroendocrine tumour, a gastrinoma. In contrast to sporadic gastrinomas that occur predominantly in the pancreas, in MEN-1 most of these tumours reside in the duodenum [1]. Duodenal gastrinomas are small, usually below 1 cm in diameter, multiple and show a preference for the proximal duodenum. Pancreatic gastrinomas associated with MEN-1 are very rare [2], as are other uncommon extra-pancreatic, extra-duodenal locations that have been associated with gastrinomas.

## 2. Pathogenesis and Classification

Independent of the tumour location, gastrinomas are well differentiated endocrine carcinomas. Recently, a specific sequence of events has been suggested to occur during tumorigenesis in gastrinomas due to multiple endocrine neoplasia [3,4]. In MEN-1 distinctive proliferative hyperplastic gastrin cells in the non-neoplastic duodenum are interpreted as multicentric precursor lesions. Their spectrum of proliferative changes is compared to those seen in entero-chromaffine-like (ECL)-cells in chronic atrophic gastritis. The first step is supposed to be diffuse hyperplasia of gastrin-positive cells, followed by linear hyperplasia, *i.e*., chains of more than five gastrin-positive cells and more than two chains per mm. With further proliferation micronodular (30–50 µm) hyperplastic lesions, *i.e*., nodules of more than 5 gastrin-positive cells within glands or crypts of the mucosa followed by enlarged nodules (>90–210 µm) of gastrin-positive cells with solid architecture have been demonstrated. These hyperproliferative and hyperplastic lesions were found only in patients with MEN-1, but were absent in patients with sporadic, not MEN-1 associated duodenal gastrinoma. The occurrence of gastrin cell hyperplasia at various sites in the duodenal mucosa explains the multifocality of gastrinomas in MEN-1. It is however unclear whether additional stimuli, besides the MEN-1 mutation are responsible for the development of hyperplastic lesions. As the next step in the tumorigenesis of MEN-1 associated gastrinomas micro-invasive lesions, small clusters of gastrin-positive cells in the lamina propria between the glands, resulting in micro-tumours (>250 µm) with trabecular growth pattern and fibrosis have been described. In MEN-1 the heterozygous germ line mutations of the MEN-1 gene, a tumour-suppressor gene, are by themselves insufficient for tumour development. According to the two-hit hypothesis of tumour development by Knudson additional somatic inactivation of the wild-type MEN-1 allele on chromosome 11q13 has to occur. This may lead to a loss of a part or all of chromosome 11, termed loss of heterozygosity. By using a combination of fluorescence *in situ* hybridisation and immuno-fluorescence techniques, Anlauf *et al*. were able to simultaneously detect allelic deletion and hormone expression in individual duodenal cells [3]. Different tumours from one patient showed different patterns of loss of heterozygosity, suggesting that each gastrinoma arises as a result of an independent second hit. In contrast none of the hyperplastic lesions was found to be positive for loss of heterozygosity. These observations enabled the authors to conclude that the hyperplastic lesions, suggested to be pre-neoplastic, retain both MEN-1 alleles and that allelic loss or other mutation in the wild-type allele inactivating the gene, is responsible for the final development of neoplastic lesions. It still remains to be analysed which factors are responsible for loss of heterozygosity in these individual cells.

To summarize, MEN-1 associated duodenal gastrinomas are preceded by gastrin-positive cell hyperplasia. Allelic deletion in these cells results in multifocal gastrin positive neoplastic lesions.

MEN-1 associated duodenal gastrinomas show a trabecular and pseudo-glandular pattern; immuno-histochemically they express gastrin and occasionally somatostatin. Interestingly only about 50% of these tumours are functionally active *i.e*., associated with a Zollinger-Ellison syndrome and thus are gastrinomas per definition [2]. They are characterised according to the WHO classification (i) as well differentiated neuroendocrine tumours, *i.e*., benign, if non-angioinvasive and below 1 cm in size or (ii) benign or low grade malignant, if confined to the mucosa-submucosa with or without angioinvasion and less than 1 cm in size. With invasion of the muscularis propria and beyond or the development of metastases they are characterized as well differentiated neuroendocrine carcinomas. Tumours with a high grade malignancy are termed poorly differentiated neuroendocrine carcinomas. Recently a TNM classification has been suggested for duodenal tumours by the European Neuroendocrine Tumour Society (ENETS) [5].

## 3. Clinical Presentation

The penetrance of duodenal gastrinomas in MEN-1 lies between non-functioning pancreatic tumours and insulinomas. Twenty to 40% of patients, between 50 and 70 years of age, are diagnosed with a gastrinoma [6]. Data from the NIH series indicate a slightly earlier diagnosis with a maximum occurring at 40 years, preceded by symptoms of a Zollinger-Ellison syndrome by 5–7 years. In comparison to sporadic gastrinomas, gastrinomas in the setting of MEN-1 are diagnosed 10 years earlier and the primary is more often located in the duodenum. No other lesion than the primary was found at the time of diagnosis in 9% of the patients with MEN-1 as compared to 27% of those with sporadic gastrinomas [7]. While recent data localize most MEN-1 related gastrinomas within the duodenum [2,8], earlier studies reported primary lymph node gastrinomas and significantly more pancreatic gastrinomas than today. This may be due to the high frequency (60 to 80%) of large regional lymph nodes metastases at the time of diagnosis [9,10]. These were either misinterpreted as primary lymph node gastrinomas due to the very small duodenal lesions that have escaped detection in patients with MEN-1, or were possibly considered as pancreatic tumours, especially if they were located at the upper margin of the head of the pancreas [11]. Duodenal gastrinomas may metastasize to the liver in about 10% of the cases, but this occurs rather late in the course of the disease. Fast-growing poorly differentiated and metastasizing duodenal gastrinomas are extremely rare [1,12,13].

The percentage of patients with gastrinomas as part of the MEN-1 syndrome is about 23% (10%–48%) in ten large series compiled by Berna and co-workers [7]. Most patients present with long-standing upper abdominal pain (66%), diarrhoea (76%), heartburn (52%), nausea (38%) and vomiting (24%) or weight loss (12%) similar to patients with sporadic gastrinoma [7,14]. Due to the notion that Zollinger-Ellison syndrome complications occur slightly less in MEN-1 compared to sporadic gastrinomas, the number of patients with bleeding as the presenting symptom of Zollinger-Ellison syndrome was significantly lower (12% *vs*. 27%) in MEN-1 gastrinomas patients [14]. The widespread use of proton-pump inhibitors may mask the classical symptoms of Zollinger-Ellison syndrome and delay the diagnosis of a gastrinoma [15]. Associated nephrolithiasis [7] occurs more often in MEN-1 patients and cutaneous lesions (angiofibromas and collagenomas) have a sensitivity of 75% and specificity of 95% to predict the presence of MEN-1 in patients with Zollinger-Ellison syndrome [16]. Interestingly, the prevalence and probability of Zollinger-Ellison syndrome in MEN-1 is sex-related, with a probability of developing Zollinger-Ellison syndrome at the age of 60 years of 55% in men and 33% in women [17].

## 4. Diagnosis

### 4.1. Biochemical Diagnosis

The diagnosis of Zollinger-Ellison syndrome is made by demonstrating an increased gastrin concentration in fasting patients in the presence of proven hyperchlorhydria. In a large prospective investigation in 309 patients with Zollinger-Ellison syndrome (including 30% MEN-1 patients), Berna *et al*. compared their findings with 2,209 cases from the literature [7]. Both, normal or very high concentrations (>100-fold normal) of gastrin concentrations in fasting patients were uncommon (0.3%–3% and 4.9%–9%, respectively). Forty to 60% of gastrinoma patients in the fasting state had a gastrin concentration below 10 times normal and this overlaps with gastrin concentrations seen in Helicobacter pylori infection. No difference could be detected between sporadic and MEN-1 associated gastrinoma patients. This increase of the gastrin concentration in fasting patients overlaps with hypergastrinaemia seen in fasting patients with idiopathic peptic disease or gastro-oesophageal reflux disease. Furthermore an increased gastrin concentration in fasting patients is observed in patients with pernicious anaemia or on proton pump inhibitor medication. However in both conditions gastric acid secretion is reduced in contrast to gastrinoma patients. In 20%–30% of these patients the gastrin concentration can be up to five times normal, comparable to the gastrin concentration seen in 60% of Zollinger-Ellison syndrome patients.

As expected increased basal acid output, tumour location (pancreas > duodenum), tumour size (large > small) and extent (liver metastases > local disease) all were positively correlated to the basal gastrin concentration.

Proton pump inhibitors have to be stopped at least 1 week prior to the determination of the gastrin concentration and this may pose a serious problem in terms of pain and risk of bleeding in symptomatic patients [7]. While histamine H2 receptor antagonists are needed in higher doses to achieve symptom control in Zollinger-Ellison syndrome compared to idiopathic ulcer disease, this is not the case with proton pump inhibitors. Thus, the use of proton pump inhibitors can delay the diagnosis of Zollinger-Ellison syndrome because of symptom control with conventional dosage.

Hypercalcaemia (due to hyperparathyroidism in MEN-1 patients, which predeces the manifestation of gastrinoma in most patients) has been shown to increase the serum gastrin concentration in fasting patients with Zollinger-Ellison syndrome. However, in the NIH series no correlation between hypercalcaemia and the gastrin concentration in fasting patients was observed, probably due to the low tumour load in MEN-1 patients.

Hypochlorhydria will increase gastrin secretion. Patients with atrophic gastritis, pernicious anaemia, post-vagotomy patients or patients on proton pump inhibitors can be excluded due to the high intragastric pH or low basal acid-output. On the other hand patients with hyperchlorhydria and hypergastrinaemia due to Helicobacter pylori infection, antral G-cell hyperfunction, short bowel syndrome or those with renal failure have to be differentiated from those with Zollinger-Ellison syndrome.

Thus, while the gastrin concentration in fasting patients with proven hyperchlorhydria is increased, in most Zollinger-Ellison syndrome patients functional testing is recommended due to the large overlap with other pathological conditions with hypergastrinaemia. Gastrin stimulation tests use either secretin or calcium to demonstrate a pathological increase in gastrin. A meal stimulation test is no longer used due to its insufficient specificity to diagnose a Zollinger-Ellison syndrome. Using a delta gastrin of 200 pg/mL, the secretin test has a sensitivity and specificity of 82% and 100% for the diagnosis of Zollinger-Ellison syndrome. The sensitivity can be increased to 94%, if a delta gastrin of 120 pg/mL is used [18]. For the calcium infusion test the corresponding numbers are 54% and 100%, respectively for a delta gastrin concentration of 396 pg/mL, while a 50% increase of the gastrin concentration after calcium stimulation resulted in a sensitivity and specificity of 78% and 83%. In those Zollinger-Ellison syndrome patients with a negative secretin test the calcium infusion test is positive in 38%–50% of the patients. Due to potential complications the calcium stimulation test is rarely used in most centres. Interestingly, there is no correlation of the secretin-stimulated gastrin response with the calcium-stimulated response. While the calcium stimulated increase in gastrin secretion has been shown to be due to a calcium sensing receptor [19], the secretin-stimulated responses have to rely on the presence of secretin receptors. It has been demonstrated that the secretin response is significantly related to the presence and density of secretin receptors in gastrinoma cells [20].

The diagnosis of Zollinger-Ellison syndrome may be further impeded due to technical difficulties with the determination of the correct gastrin concentration. Gastrin circulates not as a single molecule but as several peptides of various lengths and amino acid modifications. Moreover circulating gastrins in Zollinger-Ellison syndrome may deviate from that in healthy controls. Rehfeld *et al*. demonstrated that seven of twelve commercially available assays (radioimmunoassay and enzyme-linked immunosorbent assays) failed to accurately measure the plasma gastrin concentration [21].

### 4.2. Tumour Localisation

Once the diagnosis has been biochemically established by fasting and stimulated gastrin concentration, localization and staging of the tumour are mandatory. Here results differ considerably for patients with sporadic or MEN-1 associated tumours. Conventional imaging studies localize 10%–40%, angiography 20%–50% and somatostatin receptor scintigraphy 60%–70% of sporadic pancreatic gastrinomas. Somatostatin receptor scintigraphy is comparable to all conventional studies combined and changes the management in 15%–45% of the patients. However, sensitivity is dependent on tumour size and tumours below 1 cm are missed in about 50% of cases. As gastrinomas in MEN-1 are mostly very small duodenal lesions they are frequently missed [22]. In a prospective study in 80 patients with Zollinger-Ellison syndrome, with 22% of the patients with an associated MEN-1, somatostatin receptor scintigraphy was the most sensitive method, but had no influence on long-term survival and intra-operatively 33% additional lesions were found [23]. The guidelines for the diagnosis of MEN-1 [24] explicitly state that somatostatin receptor scintigraphy still lacks full evaluation in MEN-1. Thus, due to the small size of duodenal gastrinomas in MEN-1, these will mostly be missed by somatostatin receptor scintigraphy or conventional imaging. The highest chance for detecting these tumours will be by using multi-slice CT scanners. This technique is superior to state of the art MRI, due to its potential for acquiring an entire anatomic region continuously without gaps [25,26]. Endoscopic ultrasound, while highly effective for the detection of small pancreatic lesions fails to localize tumours in the duodenal wall. Intraoperative evaluation of the duodenum is still superior to preoperative imaging techniques, especially so when intraoperative ultrasound combined with duodenotomy are used [27].

It can be safely assumed that after a biochemical diagnosis of Zollinger-Ellison syndrome in MEN-1, multiple small tumours are probably located in the duodenum. At surgical exploration duodenotomy is essential to detect up to one-half of duodenal tumours. Intra-operative transillumination of the duodenum is frequently used to help identify the site for the duodenotomy. Intra-operative ultrasound should be routinely used to assess and identify pancreatic lesions [22].

Diagnostic imaging in gastrinoma associated with MEN-1 should preferentially aim to diagnose metastatic disease. Lymph node metastases occur in 45% to 85% of duodenal gastrinomas in MEN-1 [28]. The sensitivity and specificity of somatostatin receptor scintigraphy for lymph node metastasis as well as liver metastasis is high and somatostatin receptor scintigraphy can be highly recommended. However its sensitivity is dependent on tumours size, thus somatostatin receptor scintigraphy will miss up to 50% of small gastrinoma lesions <1 cm [29].

Metastatic disease does influence therapeutic decisions. When additional non-functioning pancreatic neuroendocrine tumours are diagnosed, any lesion in the liver can either be a metastasis of the pancreatic tumours or the gastrinoma. The prevalence of distant metastatic disease in MEN-1 due to gastrinoma is rather low and no specific data are available for the sensitivity of conventional imaging. The frequency of liver metastases from gastrinomas in MEN-1 is less by 62% compared with patients who have sporadic gastrinomas [28]. Hepatic metastatic lesions can be detected with state of the art equipment starting from 1 mm in size [26]. Yet still this does not clarify if the metastasis is due to a parallel existing non-functioning neuroendocrine pancreatic tumours or the duodenal gastrinoma. Intraoperative selective intra-arterial secretin stimulation and venous sampling of gastrin may be helpful in localising small sporadic gastrinomas. However, the procedure is not easy to perform and experience is limited, especially in patients with MEN-1.

## 5. Therapy

Therapy in MEN-1 associated gastrinoma aims at the treatment of (i) acid hypersecretion, (ii) the gastrinoma and (iii) the treatment of gastric neuroendocrine tumours type 2.

### 5.1. Medical Treatment of Acid Hypersecretion

Histamine H2-inhibitors (cimetidine, ranitidine, famotidine) have been substituted due to the need of frequent and high dosing required to control acid hypersecretion. The H1-K1 ATPase inhibitors, like omeprazole, lansoprazole, or pantoprazole, are now the drugs of choice. Acid secretion can be controlled in all patients due to the long duration of action and potency of the drugs. Most patients will need dosing once or twice and only occasionally three times a day. Acid secretion can be successfully controlled during long-term therapy with a mean omeprazole dosage of 65 mg per day or equivalent dosing of other PPI. Theses drugs are well tolerated and side effects are rare. As hypercalcaemia due to hyperparathyroidism in MEN-1 patients may exacerbate acid secretion, these patients may need higher doses of proton pump inhibitors. It is recommended to additionally address therapeutic options for hyperparathyroidism.

It should, however, not be assumed that an asymptomatic patient has adequate control of gastric acid secretion [30]. Thus, to establish the correct dosage of proton pump inhibitors acid secretion has to be determined by either measuring basic acid output 1 h prior to the next dose of anti-secretory drug or by 24-h ambulatory pH-metry. If acid secretion is suppressed to 0.10 mU/h, or 0.5 mU/h in patients with previous gastric resection or severe reflux, peptic ulcers will heal and further peptic complications will be prevented [31,32,33,34]. As has been already discussed at the diagnostic section, the hypercalcaemia of hyperparathyroidism increases gastrin secretion and, by a mechanism unknown, reduces the sensitivity of acid secretion to anti-secretory medication. Successful parathyroidectomy reduces basic acid output, gastrin secretion in fasting patients and increases the sensitivity towards anti-secretory medication allowing to reduce the dosage of anti-secretory drugs [34]. In patients with Zollinger-Ellison syndrome with MEN-1 and hypercalcaemia omeprazole 40 mg *b.i.d*. or equivalent is recommended as a staring dose and followed by a reduction to 20 mg *b.i.d*. if possible.

### 5.2. Surgical Therapy of the Gastrinoma

There has been no general recommendation for the therapy of the primary in MEN-1 associated Zollinger-Ellison syndrome. Due to the recent notion that gastrinoma in MEN-1 is a multiple tumour disease, and that most primaries are located in the duodenum, all recommendations referring to pancreatic tumours may be obsolete. The cure rate for gastrinomas within the setting of MEN-1 has been very low and this may be due to treatment strategies, which aimed at pancreatic tumours [35,36,37,38]. Further complicating the process of therapeutic decision making is the paucity of prognostic data and the synchronous existence of the gastrinoma and malignant non-functioning pancreatic neuroendocrine tumours. It is therefore difficult to discern the effect of the gastrinoma on morbidity and mortality. Thus controversies exist concerning almost every aspect of surgical therapy in gastrinoma in patients with MEN-1. Should patients controlled by proton pump inhibitors be operated at all? If surgery is considered necessary what is the optimal time-point? Should patients be operated at the diagnosis of Zollinger-Ellison syndrome or after the tumour has reached a predefined size? Should surgery be performed to control pancreaticoduodenal disease by preventing the development of metastasis? Should surgery aim at cure from Zollinger-Ellison syndrome and thus use aggressive techniques or should only individual tumours be operated on. What kind of surgery should be performed?

Overall, data compiled by Jensen demonstrate that long-term survival after diagnosis is considerably longer in patients with MEN-1 associated gastrinoma (93% at 15 years) compared to patients with sporadic gastrinoma (68% at 15 years) [34]. The analysis of eighty-one patients with MEN-1 and Zollinger-Ellison syndrome reported 100% 15 year survival with small (<2.5 cm) pancreatic tumours, as well as 100% survival in those with a single, surgically removed tumour <6 cm, but only 89% survival in those with surgery for more than two tumours or one tumour larger than 6 cm [39]. The presence of lymph node metastases does not influence, but liver metastases will shorten survival [27]. Due to the fact that liver metastases due to gastrinoma are rare in patients with MEN-1 compared to sporadic gastrinoma (2% *vs*. 21%), the percentage of patients that will develop more aggressive disease is not known. However, it has been shown that liver metastases occurred in 3% of Zollinger-Ellison syndrome patients treated surgically compared to 23% of those with only medical therapy [40]. A recent review covering 72 patients with gastrinoma (N = 15, 22% MEN-1) gave a median survival of 6.6 years. Two of 15 deaths were due to metastatic gastrinoma, with no gastrinoma related death in the MEN-1 population [41]. Goudet *et al*. investigated the risk of death according to lesions in a large cohort of 758 MEN-1 patients and found that the presence of a gastrinoma increased the risk of death (Hazard ratio 1.89, *p* = 0.022). The Hazard ratio was lower than for non-functioning pancreatic tumours (Hazard ratio 3.43, *p* = 0.001), but higher than the non-significant risk due to pituitary, bronchial tumours or insulinomas [42]. In addition considering that metastatic disease from non-functioning pancreatic tumours may be the major determinant of long-term survival, the importance of surgical therapy for gastrinoma in MEN-1 is further reduced.

Is aggressive surgery for gastrinoma without metastases justified? A retrospective analysis of surgery for Zollinger-Ellison syndrome in twelve MEN-1 patients using different surgical approaches over a period from 1970 to 2008 failed to demonstrate biochemical cure in any of the patients treated [43]. In a recent study 11 patients with Zollinger-Ellison syndrome as part of a MEN-1 syndrome were analyzed prospectively. Cure of Zollinger-Ellison syndrome was defined as a normal gastrin concentration in fasting patients and a negative secretin test. Seven of the eleven (77%) gastrinoma patients were biochemically cured after a median follow-up for 123 months (38–215 months). Four of these seven patients cured by surgery underwent pylorus-preserving pancreatico-duodenectomy. In contrast, four of the eleven patients with recurrent Zollinger-Ellison syndrome underwent local excision of duodenal gastrinomas as the initial procedure. None of these four patients had developed metastatic disease [38]. The authors therefore argue for a more aggressive therapeutic approach in duodenal-pancreatic tumours associated with MEN-1. Their objective is to detect and remove potentially malignant tumour in asymptomatic patients before malignant transformation, in an effort to reduce the death rate in those patients who undergo early surgery. Data from Skogseid and other groups support this hypothesis [38,44,45].

The high rate of post-operatively normalised basal and stimulated gastrin concentration (77%) is probably related to the use of pylorus-preserving pancreatico-duodenectomy and this is confirmed by others [1,46,47,48,49,50,51,52,53,54]. Pylorus-preserving pancreatico-duodenectomy may therefore be justified if there is a biochemical diagnosis of Zollinger-Ellison syndrome in MEN-1 and the source of gastrin can be regionalized by preoperative selective arterial secretin injection to the head of the pancreas [27,55]. Arguing against this approach are the difficult nature of this procedure, a possible high rate of morbidity and mortality, the unknown long-term consequences of pylorus-preserving pancreatico-duodenectomy as well as the problem of possible reoperation that may be rendered more difficult after pylorus-preserving pancreatico-duodenectomy than after duodenal excision or distal pancreatectomy.

A more conservative surgical approach has been to interfere only after a diagnosed lesion suggested to be a gastrinoma has surpassed a given diameter. Recommendations depend on the estimated risk for the development of metastatic disease and range from lesions larger than 1 cm, 2.5 cm or 3 cm [24,34]. The procedure most often used in these patients has been pancreatico-duodenectomy. However, Gibril and co-workers could not confirm the relationship between the incidence of liver metastases and the size of the primary tumour [56]. In addition the number of patients with both duodenal and pancreatic gastrin secreting tumours, justifying pancreatico-duodenectomy, is probably rather low [57].

A recent overview on the results of pancreatico-duodenectomy for gastrinoma in MEN-1 indicated a high cure rate (77%–100%). However most cited reports included only one or two patients, only two studies had six and thirteen patients, respectively [28].

A new option may be pancreas-preserving total duodenectomy that has been recently reported by Imamura and co-workers [57,58,59] in patients with multiple gastrinomas as part of MEN-1 syndrome. This challenging new technique awaits further investigations before it can be used as an alternative to pylorus-preserving pancreatico-duodenectomy.

In summary, no definite recommendations for surgical therapy are possible at this time for patients with MEN-1 related gastrinomas. Due to the new patho-physiological insight into the development of gastrinoma in MEN-1 with multiple very small tumours in the duodenum and a rare tumour manifestation in the pancreas Pylorus preserving pancreatico-duodenectomy may be the adequate procedure. All surgery should be performed according to oncological criteria, *i.e*., the inclusion of lymph node resection. However, in patients with additional non-functioning neuroendocrine pancreatic tumours an individualized approach with resection of pancreatic tumours is probably indicated as part of a strategy to prevent the development of hepatic metastases. This approach may be independent of the size of the pancreatic lesions.

In contrast in the patient with a sporadic gastrinoma the indication for surgery is given and the type of surgery is dictated by the localisation of the tumour and the necessary extend of surgery.

## 6. Treatment of Metastasized Gastrinoma

The most important predictor of poor survival is the presence of hepatic metastases. There are no controlled surgical trials for curative surgery in metastatic gastrinoma. Most data reported stem from mono-centric retrospective analysis in patients with functional/non-functional pancreatic neuroendocrine tumours. Major hepatic surgery with a curative approach may be justified if all or at least 90% of tumour tissue can be removed. Norton and co-workers report their results in a study with 19 gastrinoma patients. In 17 patients curative resection was performed and 5-year survival was 85%. During follow-up of 5.5 years five patients remained disease-free [60]. However, inclusion bias may have influenced the outcome of this investigation.

Recently peptide-radio receptor therapy has become available. The use of ^90^Yttrium- or ^177^Luthetium-labelled somatostatin analogues offers combination therapies to further improve outcome [61,62]. It has been suggested that peptide-radio receptor therapy is especially effective in metastasized gastrinoma with partial remission in 47% of patients with gastrinomas, insulinomas or vipomas [63]. A recent publication on the outcome of peptide-radio receptor therapy (^90^Yttrium- or ^177^Lutetium-DOTATOC) in eleven sporadic gastrinomas showed complete response in one (9%) patient, partial tumour response in 5/11 (45%), and tumour stabilization in 5/11 (45%) patients, respectively. In seven patients the anti-tumour effect persisted for 14 months, while four patients died of disease with a mean survival time after the last peptide-radio receptor therapy of 14 ± 6.9 months [64]. No prospective, randomized investigations referring to one specific tumour entity in the context of MEN-1 are available.

Octreotide therapy has been investigated in malignant gastrinomas but no data are available for its effectiveness in the setting of MEN-1. In seven of 15 patients (47%) tumour stabilization was demonstrated, and tumour size decreased in one patient (6%). The mean duration of response was 25.0 ± 6.1 months (range, 5.5–54.1 months). During follow-up (range, 4–8 years), 25% of the responders died compared with 71% of the non-responders, a difference that approached statistical significance (*p* = 0.10) [64]. Alternatively systemic chemotherapy with streptozotocin and 5-fluorouracil or doxorubicin can be offered to the patients [34,65].

## 7. Gastric Neuroendocrine Tumours Type 2

Gastric neuroendocrine tumours are currently classified according to their differentiation as well differentiated tumours, mainly composed by enterochromaffine-like cells or gastrin-producing cells. Subtyping these enterochromaffine-like cell tumours (carcinoids) classifies those associated with chronic atrophic gastritis as type 1, those associated with hypertrophic gastropathy due to Zollinger-Ellison syndrome with MEN-1 as type 2 tumours [66]. Gastric neuroendocrine tumours develop in 13%–30% of patients with Zollinger-Ellison syndrome and MEN-1. The mean age at diagnosis is 50 years [67,68]. Hypergastrinaemia is associated with entero-chromaffine-like cell hyperplasia and is seen in 90% of Zollinger-Ellison syndrome patients with MEN-1. Loss of heterozygosity at the MEN-1 locus on chromosome 11q has been clearly established in these type 2 gastric neuroendocrine tumours in contrast to type 1 gastric neuroendocrine tumours that are related to hypergastrinaemia due to atrophic gastritis [69,70,71]. Gastric neuroendocrine tumours type 2 occur in equal frequencies in both sexes, 70% are smaller than 1.5 cm, are multicentric in 90% and show invasion of the gastric wall limited to the mucosa and submucosa [67]. Metastases to lymph nodes occurred in 30% and distal metastases in 10%. However, only one patient’s death was related to the course of gastric neuroendocrine tumours type 2. While no definite recommendation can be made regarding the therapy of theses tumours, it seems justified to take a more conservative approach, following and treating these patients endoscopically as long as the lesion is below 1 cm. Whether a more aggressive approach with surgical treatment for those tumours with a size between 1 to 2 cm, or even gastrectomy in tumours larger than 2 cm is justified is currently unclear [67,72]. In addition there are no data on the effect of surgical cure, *i.e*., abolishment of hypergastrinaemia by surgical treatment of duodenal gastrinomas, on growth and progression of gastric neuroendocrine tumours type 2.

## 8. Conclusions

It has been demonstrated recently that gastrinomas associated with MEN-1 are preferentially located in the duodenum, arise from gastrin cell hyperplasia, so-called precursor lesions that develop to micro-tumours after loss of heterozygosity. The diagnosis of MEN-1 is made biochemically by determination of the fasting and secretin-stimulated gastrin concentration. Clinically hypergastrinaemia results in Zollinger-Ellison syndrome and signs and symptoms are comparable to sporadic gastrinoma. Proton pump inhibitor medication is the therapy of choice to control acid secretion in virtually all patients. Due to the small size of the primaries imaging fails to demonstrate most of these tumours, while lymph node and liver metastases may be demonstrated by somatostatin receptor scintigraphy and CT. The classical surgical approach by pancreatico-duodenectomy failed to cure most of these patients due to a possible misinterpretation of pancreatic tumours as gastrin secreting primaries. After localization of gastrin secretion to the head of the pancreas pylorus preserving pancreatico-duodenectomy has been suggested after biochemical diagnosis of Zollinger-Ellison syndrome, with a biochemical cure rate in about two-thirds of the patients. However, as most patients will harbour additional non-functioning neuroendocrine pancreatic tumours surgical strategies will have to be individualized. In metastatic gastrinomas treatment schedules follow those for sporadic gastrinomas.
